# Loss of genetic integrity in wild lake trout populations following stocking: insights from an exhaustive study of 72 lakes from Québec, Canada

**DOI:** 10.1111/eva.12160

**Published:** 2014-05-15

**Authors:** Eliane Valiquette, Charles Perrier, Isabel Thibault, Louis Bernatchez

**Affiliations:** 1 Département de Biologie, Institut de Biologie Intégrative et des Systèmes (IBIS), Université LavalQuébec, QC, Canada; 2 Ministère du Développement durable, de l'Environnement de la Faune et des ParcsQuébec, QC, Canada

**Keywords:** conservation genetics, fisheries management, genetic integrity, individual assignment, introgression, resilience, salmonid, stocking

## Abstract

Stocking represents the most important management tool worldwide to increase and sustain commercial and recreational fisheries in a context of overexploitation. Genetic impacts of this practice have been investigated in many studies, which examined population and individual admixture, but few have investigated determinants of these processes. Here, we addressed these questions from the genotyping at 19 microsatellite loci of 3341 adult lake trout (*Salvelinus namaycush*) from 72 unstocked and stocked lakes. Results showed an increase in genetic diversity and a twofold decrease in the extent of genetic differentiation among stocked populations when compared to unstocked. Stocked populations were characterized by significant admixture at both population and individual levels. Moreover, levels of admixture in stocked populations were strongly correlated with stocking intensity and a threshold value of total homogenization between source and stocked populations was identified. Our results also suggest that under certain scenarios, the genetic impacts of stocking could be of short duration. Overall, our study emphasizes the important alteration of the genetic integrity of stocked populations and the need to better understand determinants of admixture to optimize stocking strategies and to conserve the genetic integrity of wild populations.

## Introduction

Various anthropogenic pressures, such as overexploitation, habitats modifications and non-native wild or domesticated individuals' translocations, may have major impacts on the genetic makeup and evolutionary trajectory of wild populations, possibly resulting in demographic decline (Hutchings 2000; Coltman et al. 2003; Allendorf et al. 2008; Sharpe and Hendry 2009). This is especially true for fish, for which signs of overexploitation through commercial and recreational fishing have been observed for decades (Myers et al. 1997; Hutchings 2001; Post et al. 2002; Coleman et al. 2004; Post 2013). In addition, exploited fish populations have also been heavily stocked for decades to provide additional harvest opportunities in overexploited populations (Waples 1999; Helfman 2007). Although stocking is a very important tool to meet management goals, it may negatively impact the evolutionary potential of populations (reviewed in Araki et al. 2008; Fraser 2008; McClure et al. 2008) as well as their genetic integrity (reviewed in Laikre et al. 2010).

Mounting evidence indicates that captive reared fish released in the wild can experience a lower fitness than their wild conspecifics, even after very few generations of captivity (Ford 2002; McGinnity et al. 2003; Araki et al. 2007a, 2008; Thériault et al. 2011; Christie et al. 2012; Milot et al. 2013; but see Berejikian et al. 2009). Domestication selection seems the most likely explanation for this fitness reduction (reviewed in Araki et al. 2008) as captive reared fish experience different selection regimes than wild fish (Heath et al. 2003; Blanchet et al. 2008; Fraser 2008; Williams and Hoffman 2009; Christie et al. 2012). In addition, captive reared fish potentially adapted to captivity and distinct from the wild populations can lower stocked population fitness through introgressive hybridization (McGinnity et al. 2003; Araki et al. 2008, 2009). Outbreeding depression may then occur if the interbred offspring suffers a disruption of coadapted genes complex and/or a disruption of the interactions between genes and the environment they evolved in (reviewed in Allendorf et al. 2001; reviewed in Edmands 2007; Bougas et al. 2010; Granier et al. 2011).

Modification of neutral patterns of genetic diversity is also often associated with stocking. Stocked populations may show a loss of genetic diversity (Eldridge et al. 2009), a loss of genetic differentiation with other populations (Susnik et al. 2004; Eldridge and Naish 2007; Eldridge et al. 2009; Hansen et al. 2010; Marie et al. 2010; Lamaze et al. 2012; Perrier et al. 2013b) and a displacement of the local gene pool (reviewed in Laikre et al. 2010). Genetic integrity of locally adapted populations can also be threatened through introgressive hybridization (McGinnity et al. 2003, 2009; Bourret et al. 2011). Numerous studies documented various degrees of population and individual admixture in wild populations subjected to stocking (Susnik et al. 2004; Hansen et al. 2009; Marie et al. 2010; Dawnay et al. 2011; Karaiskou et al. 2011; Perrier et al. 2011; Lamaze et al. 2012). However, the link between stocking intensity and the extent of modification of local genetic diversity, including allelic richness and admixture, have been much less investigated (Hansen 2002; Marie et al. 2010; Perrier et al. 2011, 2013b; Lamaze et al. 2012). Moreover, despite the importance of such knowledge for population restoration, few studies attempted to predict the evolution of the genetic composition of stocked populations when stocking has ceased (but see Hansen and Mensberg 2009; Hansen et al. 2010; Perrier et al. 2013b).

Here, we assess the impact of stocking intensity on the evolution of the genetic composition of a large number of lake trout (*Salvelinus namaycush,* Walbaum 1792) populations from Québec, Canada. Lake trout is a freshwater fish native to North America and inhabiting cold-water lakes. It is long living (up to 49 years) and becomes sexually mature between 7 and 13 years and is iteroparous (Scott and Crossman 1998). A pronounced genetic structure has been documented among wild populations (Piller et al. 2005; Halbisen and Wilson 2009; Northrup et al. 2010; McCracken et al. 2013). Also, Piller et al. (2005) and Halbisen and Wilson (2009) reported noticeable impacts of stocking on the genetic integrity of some stocked populations. In the province of Québec, Canada, numerous lakes were stocked for over 40 years (about five generations) and the stocking history was exhaustively recorded, thus providing a unique context for documenting genetic changes of wild populations as a function of stocking history. Here, we specifically aim to (i) document genetic diversity and structure among unstocked lake trout populations, (ii) quantify the impacts of stocking on the distribution of genetic diversity within and among stocked lake trout populations, (iii) determine the influence of stocking intensity on admixture rates within stocked populations and on homogenization among source and targeted populations, and to (iv) investigate the evolution of the genetic composition of stocked populations after stocking has ceased.

## Materials and methods

### Study site, sample collection and stocking

**Figure 1:**
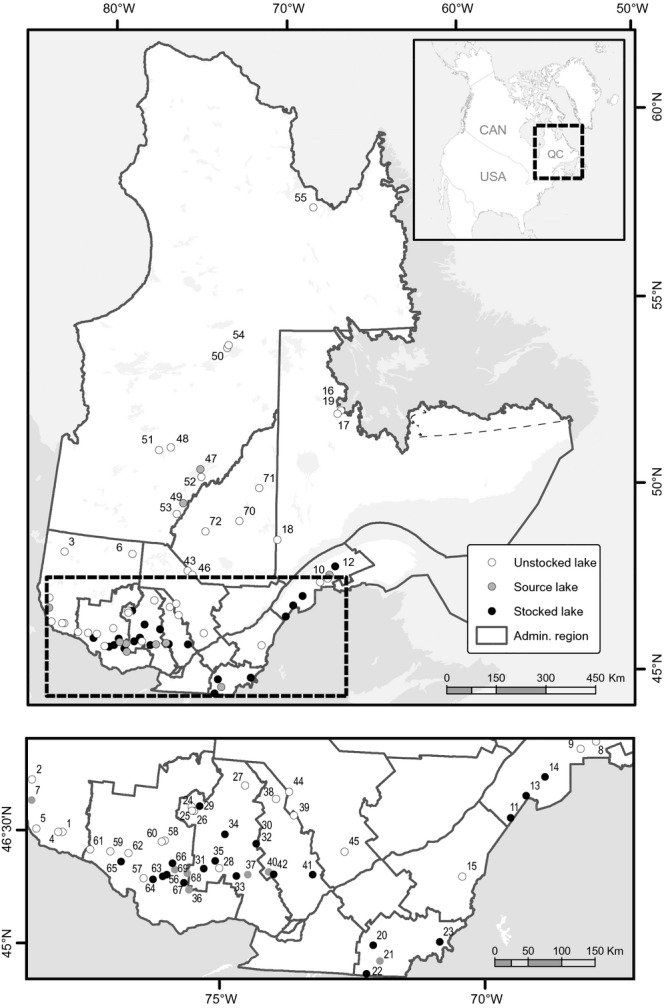
Geographical locations of sampled populations in the province of Québec, Canada (see Table 1 for more information).

A total of 3341 adult lake trout were sampled in 72 lakes from 10 independently managed and stocked administrative regions across the province of Québec, Canada (Fig. 1; Table 1, Table S4). An exception was the Chaudière-Appalaches and Estrie regions, which were both stocked using the same broodstocks. Therefore, lakes from these two regions were analyzed as if they belonged to the same region (Estrie). Adipose or pelvic fin clips were collected between 1997 and 2011 for 3003 of those individuals with the help of anglers and the provincial government. These samples were preserved in 95% ethanol. Dry scales of 338 adult lake trout from four out of 72 lakes were also collected between 1974 and 1983 (Table 1). The mean number of individuals sampled per lake was 42 (range 15–68).

**Table 1:** Sampling locations, descriptions, information, and genetic diversity indices

Region	Lake name	Lake no	Lat.	Long.	Area (ha)	Type	Stocked fish/ha	Stocking events	Stocking period	Sampled year	Sample size	*A*_R_	*H*_E_	*H*_O_	*F*_*IS*_	*N*_E_
Abitibi-Témiscamingue	Caugnawana	1	46.54	−78.31	746	U	0	0	–	2004	50	6.44	0.61	0.60	0.02	563
	Coeur (en)	2	47.18	−79.04	161	U	0	0	–	2010	27	3.14	0.41	0.42	0.01	29
	La Haie	3	48.47	−78.70	39	U	0	0	–	2008	39	3.10	0.42	0.43	−0.02	69
	Maganasipi	4	46.53	−78.39	919	U	0	0	–	2010	76	5.95	0.60	0.58	0.05	279
	Marin	5	46.54	−78.82	401	U	0	0	–	2009	24	4.38	0.50	0.49	0.04	129*
	Terrasses (des)	6	48.64	−75.92	272	U	0	0	–	2003	18	4.85	0.60	0.63	−0.02	77
	Kipawa	7	46.91	−78.99	30 044	So–S	4	6	1986–1998	2010	50	8.90	0.79	0.77	0.03	–
Bas-Saint-Laurent	Chasseurs (des)	8	48.22	−67.87	243	U	0	0	–	2000	38	5.44	0.64	0.65	0.00	71
	Côté	9	48.12	−68.18	119	U	0	0	–	2010	18	3.40	0.45	0.41	0.13	2
	Mitis	10	48.32	−67.80	1015	So–S	83	17	1989–2009	2009	50	8.22	0.75	0.81	−0.07*	–
	Est (de l')	11	47.19	−69.56	192	S	454	12	1975–2009	2010	49	8.02	0.75	0.70	0.08*	–
	Matapédia	12	48.55	−67.56	3807	S	63	20	1979–2009	2009	48	8.35	0.75	0.73	0.04	–
	Pohénégamook	13	47.49	−69.27	894	S	154	15	1982–2007	2009	48	9.09	0.79	0.79	0.02	–
	Témiscouata	14	47.75	−68.89	6682	S	53	18	1977–2006	2008	57	6.90	0.68	0.66	0.03	–
Chaudière-Appalaches	Etchemin	15	46.39	−70.49	251	U	0	0	–	2010	33	7.03	0.75	0.71	0.06	78
Côte-Nord	Bloom	16	52.83	−67.24	103	U	0	0	–	2010	45	3.69	0.53	0.52	0.02	513
	Daigle	17	52.81	−67.21	235	U	0	0	–	2010	46	3.51	0.44	0.45	−0.01	47
	Sault aux Cochons	18	49.28	−69.96	901	U	0	0	–	2009	42	5.10	0.61	0.62	0.00	224
	Webb	19	52.73	−67.36	124	U	0	0	–	2010	37	4.97	0.60	0.60	0.02	66
Estrie	Brompton	20	45.42	−72.15	1191	S	163	42	1949–2006	2010	48	9.86	0.83	0.83	0.01	–
	Massawippi	21	45.21	−72.00	1792	So–S	244	50	1952–2009	2009	68	9.30	0.83	0.82	0.02	–
	Memphrémagog	22	45.03	−72.24	9531	S	40	46	1952–2009	2009	46	9.38	0.83	0.86	−0.02	–
	Mégantic	23	45.50	−70.88	2692	S	97	45	1952–2006	2010	47	8.88	0.82	0.86	−0.05	–
Laurentides	Bondy	24	47.08	−75.85	531	U	0	0	–	2011	43	7.71	0.71	0.72	−0.01	400*
	Marguerite	25	47.03	−75.80	622	U	0	0	–	2011	50	7.58	0.72	0.72	0.01	873
	Madeleine	26	47.03	−75.84	319	U	0	0	−	2011	50	7.11	0.72	0.74	−0.02	220
	Turnbull	27	47.44	−74.85	352	U	0	0	−	2011	21	5.97	0.63	0.62	0.04	611
	Joinville	28	46.30	−75.20	259	U	0	0	−	2010	50	7.76	0.74	0.70	0.06	147
	Crevier	29	47.10	−75.70	438	S	30	5	1994−2002	2010	46	9.65	0.76	0.75	0.05	–
	Grandive	30	46.65	−74.56	137	S	124	5	1994–2002	2010	50	10.36	0.83	0.86	−0.02	–
	Cerf (du)	31	46.28	−75.50	1267	S	123	18	1957–2008	2010	58	10.20	0.81	0.82	−0.01	–
	Grand Froid	32	46.67	−74.52	215	S	60	6	1972–1983	2010	26	7.67	0.74	0.71	0.05	–
	Labelle	33	46.22	−74.86	738	S	89	24	1952–2007	2010	48	10.44	0.84	0.84	0.01	–
	Pérodeau	34	46.76	−75.15	238	S	75	8	1958–2005	2010	50	6.74	0.67	0.67	0.00	–
	Pimodan	35	46.39	−75.29	357	S	135	13	1955−2008	2010	42	11.15	0.86	0.87	−0.01	–
	Poisson Blanc	36	45.97	−75.74	8521	So−S	26	11	1972–2001	2010	16	9.78	0.79	0.82	−0.02	–
	Tremblant	37	46.25	−74.64	945	So−S	412	29	1942–2008	2010	39	10.16	0.83	0.82	0.02	–
Lanaudière	Tourbis	38	47.29	−74.21	1401	U	0	0	–	2010	39	4.26	0.57	0.60	−0.04	94
	Devenyns	39	47.09	−73.84	2163	U	0	0	–	2010	50	6.80	0.70	0.70	0.01	110
	Archambault	40	46.31	−74.24	1380	So−S	35	8	1950–2008	1997	28	6.40	0.67	0.67	0.02	–
	Maskinongé	41	46.32	−73.38	1018	S	111	24	1955–2004	2010	20	5.77	0.65	0.66	0.01	–
	Ouareau	42	46.28	−74.14	1492	S	36	8	1992–2004	2010	50	8.37	0.74	0.74	0.02	–
Mauricie	Goélands (aux)	43	48.31	−73.60	212	U	0	0	–	2009	15	5.00	0.54	0.60	−0.07	58
	Mondonac	44	47.40	−73.96	2313	U	0	0	–	2007	47	7.29	0.71	0.67	0.07*	51
	Piles (des)	45	46.65	−72.79	401	U	0	0	–	2008	39	6.14	0.65	0.64	0.03	98*
	Rita	46	48.22	−73.40	179	U	0	0	–	2009	24	5.15	0.57	0.55	0.05	21*
Nord-du-Québec	Mistassini	47	51.11	−73.36	211343	So	0	0	–	2010	46	11.54	0.83	0.77	0.08*	425
	30655	48	51.64	−74.73	420	U	0	0	–	1997	45	9.96	0.80	0.78	0.03	337
	Waconichi	49	50.15	−74.00	8184	So	0	0	–	2010	50	9.58	0.81	0.79	0.03	2138
	Lovely lake	50	54.47	−72.49	481	U	0	0	–	2010	34	9.09	0.78	0.79	0.00	25
	Khawashicmitch	51	51.54	−75.23	401	U	0	0	–	2009	34	6.66	0.69	0.65	0.08*	156
	Albanel	52	50.90	−73.29	40 663	U	0	0	–	2010	49	5.44	0.64	0.63	0.03	2509
	Chibougamau	53	49.84	−74.23	20 616	U	0	0	–	2002	49	10.14	0.84	0.81	0.04	1808
	des Oeufs	54	54.55	−72.43	5110	U	0	0	–	2010	50	5.87	0.67	0.67	0.01	562*
	Tasiapik	55	58.36	−68.45	26	U	0	0	–	2010	29	3.00	0.48	0.52	−0.07	48*
Outaouais	Patterson	56	46.14	−76.20	109	S	59	9	1991–2007	2011	33	9.17	0.78	0.80	−0.01	–
	Bell	57	46.06	−76.63	300	U	0	0	–	2005	40	6.46	0.65	0.61	0.07	197*
	Désert	58	46.59	−76.31	2978	U	0	0	–	1977	66	9.97	0.74	0.74	0.02	294
	Désert	58	46.59	−76.31	2978	U	0	0	–	2010	30	6.59	0.70	0.72	−0.01	113*
	St-Patrice	59	46.37	−77.33	2849	U	0	0	–	1979	63	10.21	0.74	0.69	0.09*	512
	St-Patrice	59	46.37	−77.33	2849	U	0	0	–	2010	49	7.26	0.74	0.72	0.04	121*
	Royal	60	46.57	−76.37	339	U	0	0	–	1977	66	6.91	0.64	0.63	0.02	1397*
	Royal	60	46.57	−76.37	339	U	0	0	–	2010	47	5.52	0.63	0.62	0.03	41*
	Vert	61	46.36	−77.73	176	U	0	0	–	2010	52	6.33	0.72	0.69	0.06	73
	Antoine	62	46.37	−76.99	435	U	0	0	–	2011	48	5.27	0.59	0.60	0.00	90
	Cayamant	63	46.12	−76.27	725	S	113	16	1964–2007	2011	39	9.53	0.79	0.77	0.05	–
	Dumont	64	46.06	−76.46	1772	S	28	12	1988–2005	2011	44	6.72	0.68	0.66	0.04	–
	Achigan (de l')	65	46.25	−77.10	673	S	50	7	1977–1995	2010	49	9.77	0.81	0.8	0.02	–
	Cèdres (des)	66	46.30	−76.11	793	S	34	8	1964–2005	2010	23	8.91	0.78	0.75	0.06	–
	Pemichangan	67	46.06	−75.85	1544	S	49	18	1973–2009	2010	50	8.61	0.79	0.78	0.03	–
	31-Milles	68	46.19	−75.81	4973	So−S	11	4	1962–1971	1974	75	12.66	0.79	0.77	0.04	–
	31-Milles	68	46.19	−75.81	4973	So−S	33	12	1962–1982	1983	68	14.07	0.80	0.82	−0.01	–
	31-Milles	68	46.19	−75.81	4973	So−S	67	23	1962–1995	2003	36	9.88	0.80	0.78	0.03	–
	Blue Sea	69	46.22	−76.05	1437	So−S	190	24	1972–2009	2010	49	9.60	0.81	0.81	0.01	–
Saguenay-Lac-St-Jean	Dulain	70	49.75	−71.57	391	U	0	0	–	2010	36	5.10	0.56	0.57	0.01	119*
	Manouane	71	47.57	−74.11	46 102	U	0	0	–	2009	43	8.56	0.75	0.71	0.07*	139*
	Rond	72	49.42	−72.98	686	U	0	0	–	2009	32	4.68	0.52	0.56	−0.04	17*

We include region, lake name and number and location for each lake.

Type is the lake status as unstocked (U), stocked (S) or source (So), stocking intensity is given in stocked fish per hectare (Stocked fish/ha) and in number of stocking events (Stocking events), *A*_R_ is the allelic richness based on 15 individuals, *H*_E_ is the expected heterozygosity, *H*_O_ is the observed heterozygosity and *F*_IS_ is the inbreeding coefficient (significance is indicated by asterisk), *N*_E_ is the effective size (as estimated using a linkage disequilibrium method or with Nei estimator when marked with†).

The stocking history of each stocked lake is recorded by the provincial government since 1900 in a central database. For each stocking event, a standardized stocking form is filed, compiled in the database and carefully verified to detect potential errors. Although the database is reliable, we performed additional validation of the data for the stocked lakes used in this study for further improvements. Lakes were categorized as ‘unstocked’ when no stocking was recorded and as ‘stocked’ when at least one stocking event was documented. The mean number of stocking events in stocked lakes was 18 (range 5–50). A source lake was a lake where broodstock was collected and used to produce juveniles in captivity, which were released in wild populations, usually at age 1+. Of the 72 sampled lakes, 10 were source lakes (*n* = 432), 40 were unstocked lakes (*n* = 1600) and 22 were stocked lakes (*n* = 971). Of the 10 source lakes, two were never stocked, whereas eight were stocked with local and/or nonlocal individuals. Among lakes sampled between 1974 and 1983, three were never stocked and one was stocked with juveniles produced with its own broodstock after 1962. These samples were used to validate the temporal stability of genetic characteristics within populations never stocked with non-native individuals as contemporary samples were also collected as described above.

### DNA extraction

DNA was extracted from adipose fin and pelvic fin clip (2 mm^2^) using a modified version of Aljanabi and Martinez (1997) salt extraction method. DNA precipitation was performed with isopropanol for 30 min. After washing the pellets, we performed a centrifugation at 10 000 *g* for 10 min at 4°C. Finally, we eluted the samples in 100 *μ*L sterile dH_2_0. DNA from dried scales was extracted using the Qiagen DNeasy Blood and Tissue kit (Qiagen inc., Valencia, CA, USA) following a modified version of the manufacturer's Bench Protocol for animal tissues (spin-column protocol). Namely, we incubated samples with proteinase K at 37°C for 3 days. After having discarded flow-through (buffer AW2), we performed a second centrifugation for 1 min at 20 000 *g*. We then heated up buffer AE at 56°C and used 75 *μ*L of it to perform a first elution. We repeated the elution with a second 75 *μ*L warm AE buffer.

### DNA amplification and microsatellite genotyping

Individuals collected between 1997 and 2011 (*n* = 3003) were analyzed at 19 microsatellite loci using Qiagen Multiplex PCR Kit (Qiagen inc.) (Table S1, Supporting information). Each 10 *μ*L multiplex PCR reaction contained 5–25 ng DNA template, 5 *μ*L Qiagen multiplex reaction buffer and forward and reverse primers at different concentrations: multiplex A contained 4.5 *μ*m *SfoD75*, 3 *μ*m *Sfo308lav*, 3.5 *μ*m *Sfo226lav*, 0,8 *μ*m *SnaMSU02*, 1 *μ*m *SnaMSU03*; multiplex B contained 3 *μ*m *SnaMSU06*, 0.2 *μ*m *SnaMSU08*, 6.5 *μ*m *SnaMSU09*, 7.0 *μ*m *SnaMSU10,* 7 *μ*m *Sco202*; multiplex C contained 0.5 *μ*m *SnaMSU11*, 2 *μ*m *SnaMSU12*, 1.5 *μ*m *SnaMSU13*, 10 *μ*m *SnaMSU07*, 0.25 *μ*m *Sco215*; multiplex D contained 1 *μ*m *SnaMSU01*, 2.5 *μ*m *Sco200*, 0.3 *μ*m *Smm22*, 1.2 *μ*m *Sssp2201* (Crane et al. 2004; Dehaan and Ardren 2005; Paterson et al. 2004; Perry et al. 2005; Rollins et al. 2009; T. L. King, S. E. Julian, R. L. Coleman and M. K. Burnham-Curtis, unpublished). Amplifications were performed using a T1 Biometra thermocycler (Biometra, Kirkland, QC, Canada) with a 15 min activation step at 95°C, followed by 35 cycles of denaturation at 95°C for 30 s, annealing for 3 min at 60°C (multiplex A, B and C) or 56°C (multiplex D), extension at 72°C for 1 min, followed by a final extension at 72°C for 10 min. Individuals collected between 1974 and 1983 were genotyped with the same loci except for *Sfo226Lav*, *Sfo308Lav*, *Sco215*, *SnaMSU07* and *Sssp2201* which did not amplify or amplified poorly with old DNA template. These individuals were amplified in multiplex with the same PCR programs except for multiplex A for which we used an annealing temperature of 56°C instead of 60°C. Amplified products were migrated via electrophoresis using an ABI 3130*xl* capillary DNA sequencer (Applied Biosystems Inc.). Genescan 500 LIZ (Applied Biosystems Inc., Burlington, ON, Canada) was used as a standard to determine allele sizes, which were scored using genemapper 4.0 (Applied Biosystems Inc).

### Genetic diversity

The software micro-checker (Van Oosterhout et al. 2004) was used to assess the potential presence of null alleles and large allelic dropout. *F*_IS_ and number of alleles per locus (*A*) were estimated using fstat 2.9.3 (Goudet 2001). Allelic richness (*A*_R_) adjusted for the smallest sample size (*n* = 15) was calculated using HP-Rare 1.0 (Kalinowski 2005). We calculated mean A_R_ for unstocked and stocked populations and tested for difference using Student's *t*-test. We used a linear model to assess the relationship between A_R_ and the natural logarithm of lake area [ln(LakeArea)] for unstocked and stocked lakes. Strength of the correlation and significance were assessed with Pearson's product-moment correlation coefficient. Expected and observed heterozygosities (*H*_E_ and *H*_O,_ respectively) were obtained with genetix 4.05 (Belkhir et al. 1996). We calculated mean *H*_E_ for unstocked and stocked populations and tested for difference using Student's *t*-test. Effective population size (*N*_E_) was estimated using the linkage disequilibrium method implemented in LDNe (Waples and Do 2010) for unstocked lakes only. *N*_E_ would have been meaningless in stocked lakes due to biases resulting from individual and population admixture which increase linkage disequilibrium (Araki et al. 2007b). We used a linear model to assess the relationship between *N*_E_ and ln(LakeArea) and between *N*_E_ and *A*_R_. Strength of the correlations and significance were assessed with Pearson's product-moment correlation coefficient.

### Population genetic structure

We quantified the extent of genetic differentiation between each pair of populations using pairwise *F*_ST_ measures with fstat 2.9.3 (Goudet 2001). We tested for difference in genetic differentiation between the unstocked versus stocked lakes with fstat using the comparison among groups with 10 000 permutations. To assess the relationship between stocking intensity and genetic differentiation among source and stocked populations, we used Spearman's rank test correlation. Stocking intensity was calculated as the total number of fish stocked per hectare and as the number of stocking events (years).

We assessed the distribution of genetic diversity among administrative regions, among populations within regions, and within populations using analyses of molecular variance (amova) implemented in arlequin 3.5 (Excoffier and Lischer 2010) and tested with 10 000 permutations. To assess the influence of stocking on the distribution of genetic diversity among and within regions, three different amovas were performed. The first amova was performed with unstocked populations (*n* = 42) grouped by 10 regions (Abitibi-Témiscamingue, Bas-Saint-Laurent, Chaudière-Appalaches-Estrie, Côte-Nord, Lanaudière, Laurentides, Mauricie, Nord-du-Québec, Outaouais, Saguenay-Lac-Saint-Jean). The second amova was performed with stocked populations (*n* = 30) grouped by a subset of five regions where stocking occurred (Bas-Saint-Laurent, Chaudière-Appalaches-Estrie, Lanaudière, Laurentides, Outaouais) as the other five did not contain any stocked populations. The third amova was performed with these same five regions but with only the unstocked populations (*n* = 16) for strict comparison with the above-mentioned amova.

### Admixture analyses

Individual clustering was achieved using the Bayesian method implemented in structure 2.3.1 software (Pritchard et al. 2000). This analysis was carried out to assess the number of genetic clusters present in our data and quantify admixture proportions at both the individual and population levels among stocked and source populations. structure analyses were performed assuming an admixture model without priors. We ran the analysis for seven different lake groupings (defined below) with *k* genetic clusters from 1 to the number of lakes plus 3 (number of lakes varied from 9 to 42 according to the group of lakes considered), and with 15 replicates for each *k*. The first group was composed of all unstocked lakes only. The second to the sixth groups each represented one of the five stocked administrative regions, composed of unstocked lakes, stocked lakes, and all known source lakes used for stocking within each region. The last group was composed of the four lakes with historical and contemporary samples.

Each Structure run started with a burn-in period of 50 000 followed by 300 000 Markov Chain Monte Carlo (MCMC) steps. For each lake group considered, we selected the best *k* according to the variation of likelihood (Pritchard et al. 2000), the Δ*k* method (Evanno et al. 2005) implemented in structure harvester (Dent and vonHoldt 2012) and consistency of structure outputs. The software distruct (Rosenberg 2004) was used to plot structure outputs.

To further investigate the impact of stocking, we quantified proportions of fish belonging to local clusters, nonlocal clusters, or to a category of individuals putatively admixed between local and nonlocal populations and representing putative hybrids. For this analysis, we used lakes sampled within the five stocked regions and excluded source lakes. Following Vähä and Primmer (2006), individuals were classified as local, nonlocal and putatively admixed when their individual admixture proportion (*ind. q*-values) of the local cluster were, respectively, >0.70, <0.30, and [0.30 and 0.70]. For statistical analyses, stocked and unstocked lakes were separated into distinct groups. A chi-square test was then used to test whether differences in proportions of local, admixed, and nonlocal individuals between the unstocked and the stocked groups were significant. This test was repeated for unstocked and stocked groups within each of the five stocked regions. Chi-square was also used to test whether differences in the proportion of these individuals were significant among stocked as well as unstocked groups between regions.

### Effects of stocking intensity on introgressive hybridization

We investigated the effects of stocking intensity on population admixture (*q*-membership) in two ways using R [R Development Core Team (2012)]. Firstly, we applied a logistic regression model to investigate the link between stocking intensity and the membership of stocked populations to the local cluster (*local q*-membership). Unstocked populations were included in the regression as they were used to assess *local q*-membership when no stocking was documented. We attributed a value of ‘1’ to populations with a *local q*-membership >0.50 and a value of ‘0’ to populations with a *local q*-membership ≤0.50 as few intermediate values were found (see Results section). Secondly, we examined the link between the number of stocked fish per hectare and the membership of stocked populations to their source(s) cluster (*source q*-membership) as well as the link between the number of stocking events and the *source q*-membership using Spearman's rank test correlations. For these analyses, *source q*-membership was used, as many sources could have been used for each stocked lake.

Lastly, we assessed the effect of the time spent (in years) since the last stocking event on the populations' *source q*-membership. For this latter analysis, as stocking pressure may influences populations' *source q*-membership, we only used pairs of source and stocked populations that exchanged similar number of fish per hectare to eliminate potential bias originating from differences in stocking intensity. The only stocking intensity in number of fish stocked per hectare for which we had consistent data over the 40 years period was 17–22 fish stocked per hectare. Nine pairs of source and stocked populations corresponded to this criterion (# unstocked- # source: 11–47, 11–68, 14–10, 20–68, 22–21, 23–68, 32–48, 33–68, 42–37). For this analysis, we could not find pairs of source and stocked populations with similar number of stocking events over the 40 years period. We used Spearman's rank test correlation to assess significance of the correlations.

## Results

### Genetic diversity

Population genetic diversity indices for each population are shown in Table 1. Among the contemporary samples (collected between 1997 and 2011), 72 of 1368 permutation tests conducted in fstat revealed significant *F*_IS_ values. However, micro-checker showed that only seven of these 72 significant *F*_IS_ were associated with null alleles. At the population level, six of the 72 *F*_IS_ tests were significant. Of these, one was associated with the potential presence of null alleles (#71) and two corresponded to stocked populations (#10 and 11). For the historical samples (collected between 1974 and 1983), six of 70 permutation tests yielded significant *F*_IS_ values of which five were associated with null alleles for five different loci. At the population level, one of five *F*_IS_ was significant and associated with the potential presence of null alleles (#59).

**Figure 2:**
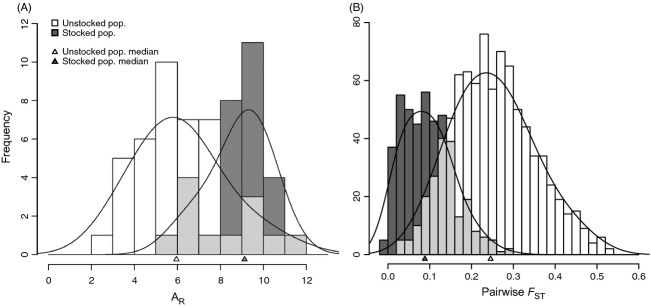
(A) Distribution of *A*_R_ value frequency for unstocked (*N* populations = 42; white bars) and stocked (*N* populations = 30; dark gray bars) populations; triangles indicate median A_R_ value for each distribution; light gray is the overlapping between the two distributions, (B) distribution of pairwise *F*_ST_ frequency among unstocked (N pairwise comparisons = 861; white bars) and among stocked populations (N pairwise comparisons = 435; dark gray bars); triangles indicate median *F*_ST_ for each distribution; light gray is the overlapping between the two distributions.

All 19 loci were moderately to highly polymorphic with the number of alleles per locus ranging from 8 to 56 with an average of 32. Over all loci and populations, a total of 613 alleles were identified. Global *A*_R_ was 11.96 over all loci and populations, while mean *A*_R_ per population was 7.29 and ranged from 3.00 to 11.54. A_R_ was significantly higher in stocked (mean *A*_R_ = 8.86 ranging from 5.77 to 11.15) compared unstocked populations (mean *A*_R_ = 6.16 ranging from 3.00 to 11.54) (*t* = −6.15; d.f. = 57.61; *P* < 0.0001; Fig 2A). The ln(LakeArea) was positively correlated with *A*_R_ among unstocked populations (*R*^2^ = 0.41; *t* = 5.44; *P* < 0.0001), but not among stocked populations (*R*^2^ = 0.03; *t* = 0.257; *P* = 0.80). Average *H*_E_ was 0.695 over all populations ranging from 0.415 to 0.856. As observed for *A*_R_, *H*_E_ was significantly higher among stocked populations (mean *H*_E_ = 0.774 ranging from 0.651 to 0.856) than among unstocked ones (mean *H*_E_ = 0.639 ranging from 0.4147 to 0.836) (*t* = −6.71; d.f. = 64.11; *P* < 0.0001). *N*_E_ varied from 2 to 2509 among unstocked populations (with a median value of 134). We detected a significant positive correlation between *N*_E_ and lake ln(LakeArea) (*R*^2^ = 0.26; *t* = 3.92; *P* < 0.001), and between *N*_E_ and *A*_R_ (*R*^2^ = 0.09; *t* = 2.25; *P* = 0.0298).

### Population genetic structure

**Figure 3:**
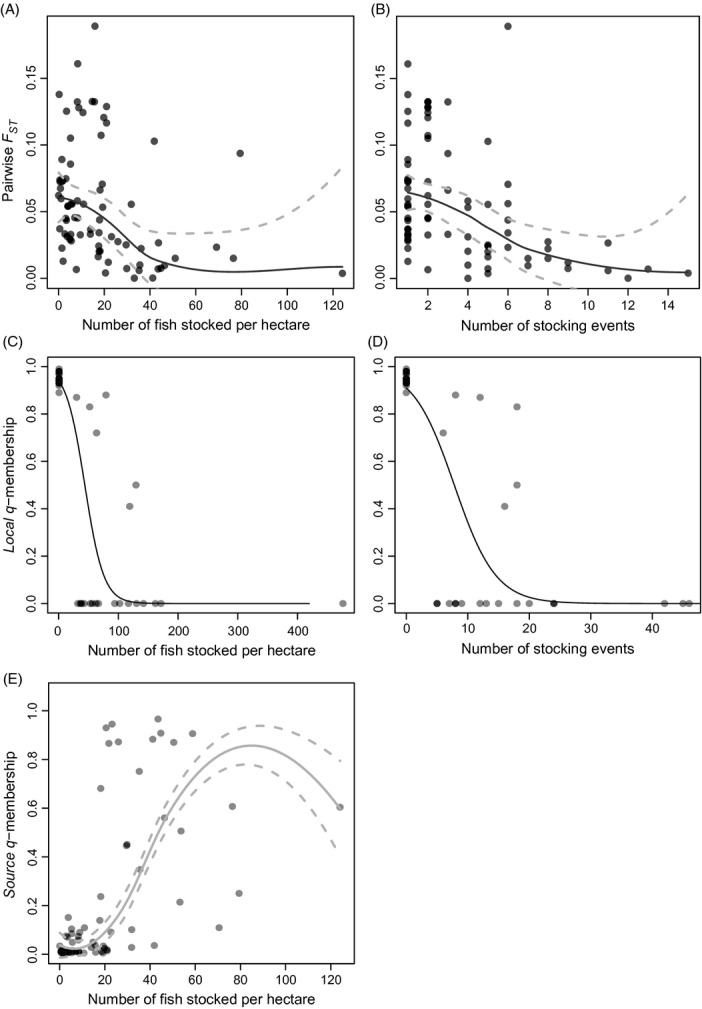
(A) Pairwise *F*_ST_ values between source and stocked populations (N pairwise comparisons = 78) as a function of stocking intensity in number of fish per hectare and B) in number of stocking event with a 0.95 confidence envelope, C) populations' membership (N pop. = 38; N ind. = 1629) to their own genetic cluster (*local q*-membership) as a function of stocking intensity in number of fish stocked per hectare and D) in number of stocking events, E) stocked populations' membership to their source populations cluster (*source q*-membership) as a function of number of fish stocked per hectare (colors) (N pairwise comparisons = 69), with a 0.95 confidence envelope.

Results showed that stocking had a profound effect on patterns of population structure. Estimates of global *F*_ST_ among the 42 unstocked populations was more than twice of that observed among the 30 stocked populations {0.227, (CI [0.197–0.260]) vs. 0.102 (CI [0.082–0.123]), *P* < 0.0001}. Pairwise *F*_ST_ values ranged from 0.025 to 0.531 among unstocked populations and from −0.016 to 0.298 among stocked populations (Fig 2B; Table S2, Supplementary material). Among unstocked populations, the variance of pairwise *F*_ST_ values was large with a median value of 0.246, but much smaller among stocked populations with a median value of 0.088 (Fig. 2B). Moreover, a highly significant negative correlation was observed between pairwise *F*_ST_ values comparing source versus stocked populations and stocking intensity, either in number of fish stocked per hectare (*ρ* = −0.432; *P* < 0.0001; Fig 3A) or in number of stocking events (*ρ* = −0.489; *P* < 0.0001; Fig 3B). amovas also revealed that genetic differentiation among populations within region was much more pronounced for unstocked populations with *F*_SC_ = 0.199 (CI [0.173–0.226]) than stocked *F*_SC_ = 0.041 (CI [0.033–0.050]) (*P* < 0.0001; Table 2). In contrast, genetic differentiation among regions was twice lower for unstocked populations with *F*_CT_ = 0.036 (CI [0.026–0.048]) as compared to *F*_CT_ = 0.064 (CI [0.050–0.078] for stocked (*P* < 0.0001; Table 2).

**Table 2:** Global genetic differentiation between populations (*F*_ST_) as calculated by fstat, among lakes within regions structure (*F*_SC_) and among regions structure (*F*_CT_; 42 populations within 10 regions for the all unstocked populations group, 16 populations within five regions for the subset of unstocked populations group and 30 populations within five regions for the stocked group) as calculated by Arlequin

	*F*_ST_ [5–95%]	*F*_SC_ [5–95%]	*F*_CT_ [5–95%]
All unstocked populations	0.227 [0.197–0.260]	0.199 [0.173–0.226]	0.036 [0.026–0.048]
Subset unstocked populations	0.184 [0.155–0.221]	0.166 [0.143–0.191]	0.029 [0.017–0.042]
Stocked populations	0.102 [0.082–0.123]	0.041 [0.033–0.050]	0.064 [0.050–0.078]

Pariwise *F*_ST_ values among historical and contemporary populations were not different from 0 except for population #59 for which a small *F*_ST_ value of 0.01 was found (Table S3).

### Admixture analyses

**Figure 4:**
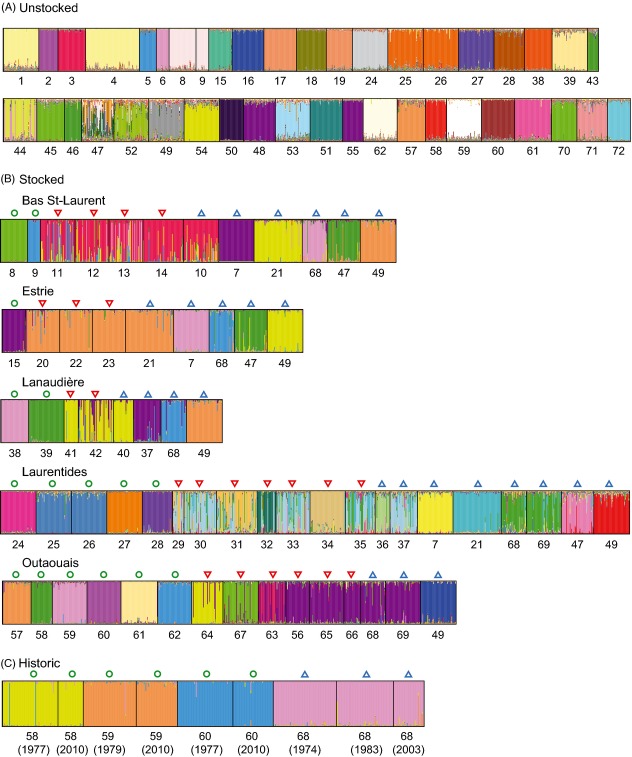
Bayesian individual clustering results with structure for A) unstocked populations (*k* = 39); (B) regions where stocking occurred, with the unstocked populations on the left, the stocked populations in the middle and the source populations used for each region on the right (Bas-Saint-Laurent for *k* = 8, Estrie-Chaudière-Appalaches for *k* = 6, Lanaudière for *k* = 6, Laurentides for *k* = 14 and Outaouais for *k* = 11); (C) historical samples compared to contemporary samples for *k* = 4. Colored columns represent proportions of membership of each individual to each cluster. Green circles represent unstocked populations, downward pointing red triangles represent stocked populations and blue triangles represent source populations.

Most genetic clusters delineated by structure corresponded to a unique unstocked lake. Among the 42 unstocked populations, *k* = 39 was the most likely number of clusters (Fig 4A). In six cases, one cluster corresponded to two lakes. Also, two lakes comprised more than one cluster. However, performing independent clustering analyses for each region, each unstocked population corresponded to a single cluster except lakes #25 and #26, which corresponded to a same cluster, and lakes #44 and #47, which corresponded respectively to two and four clusters. Lakes #25 and #26 were in fact geographically close to each other (2.7 km) and physically connected. Considering both unstocked and stocked populations on a regional scale, the best clustering solutions were *k* = 8 for the Bas-Saint-Laurent region, *k* = 6 for the Estrie-Chaudière-Appalaches region, *k* = 6 for the Lanaudière region, *k* = 17 for the Laurentides region and *k* = 11 for the Outaouais region (Fig 4B). In every stocked region, stocked populations shared various proportions of ancestry with their source(s), while unstocked populations corresponded to a unique cluster (except lakes 25 and 26; Fig. 4B; Table 3). Finally, *k* = 4 was retained in the case of the comparison of historical and contemporary samples (Fig 4C). Each unstocked population corresponded to the same unique cluster in both contemporary and historical samples.

**Table 3:** Membership of stocked populations attributed to their stocking source lakes (*source q*-membership). Underlined values represent putative undocumented stocking between lakes while italic data represent putative indirect admixture via other source lakes that were themselves stocked. Dashes indicate that stocking was not documented in our analyses

	Source lake no								
	
Stocked lake no	7	10	21	36	37	40	47	49	68
11	–	0.61	0.10	–	–	–	–	–	0.03
12	–	0.75	0.10	–	–	–		–	0.05
13	–	0.56	0.11	–	–	–	–	–	0.25
14	–	0.87	0.06	–	–	–		–	0.01
20	0.01	–	0.88	–	–	–	–	0.01	0.03
22	0.01	–	0.93	–	–	–	0.03	–	0.02
23	0.00	–	0.97	–	–	–	–	0.01	0.01
29	–	–	0.03	–	0.45	–	–	0.03	0.02
30	–	–	0.10	0.04	0.60	–	–	–	0.10
31	0.01	–	0.07	0.08	0.14	–	0.01	0.01	0.11
32	–	–	–	–	0.16	–	–	0.01	0.04
33	–	–	0.15	0.12	0.45	–	0.09	0.01	0.09
34		–	0.03	–	–	–	–	–	0.02
35	–	–	0.21	0.03	0.35	–	0.05	0.03	0.21
41	–	–	–	–	0.01	0.95	–	0.01	0.04
42	–	–	–	–	0.24	0.68	–	–	0.05
56	–	–	–	–	–	–	–	–	0.91
63	–	–	–	–	–	–	–	–	0.51
64	–	–	–	–	–	–	–	–	0.09
65	–	–	–	–	–	–	–	–	0.87
66	–	–	–	–	–	–	–	–	0.87
67	–	–	–	–	–	–	–	–	0.10

**Figure 5:**
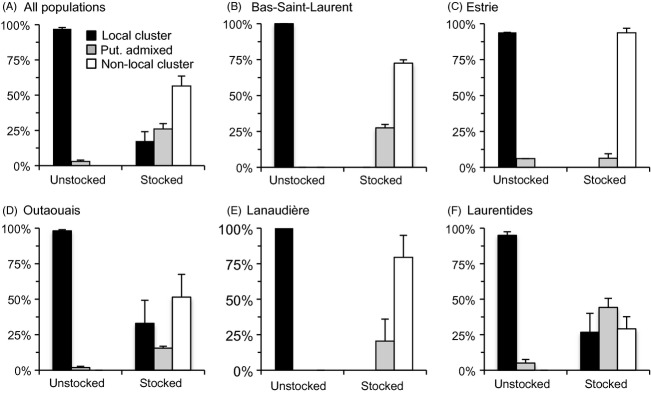
Percentages (±SE) of fish assigned to local cluster, putatively admixed category (put. Admixed) or nonlocal cluster for unstocked and stocked populations: (A) all populations sampled in stocked regions (N pop. = 38; N ind. = 1629), B) Bas-Saint-Laurent (N pop. = 6; N ind. = 258), C) Estrie-Chaudière-Appalaches (N pop. = 4; N ind. = 174), D) Outaouais (N pop. = 11; N ind. = 504), E) Lanaudière (N pop. = 4; N ind. = 159), F) Laurentides (N pop. = 12; N ind. = 534).

Admixtures analyses also revealed that stocking had a profound impact on the genetic integrity of lake trout populations. Considering the whole dataset, the proportion of fish assigned to the local cluster, a nonlocal cluster or a putatively admixed category differed between unstocked and stocked lakes (*χ*^2^_(2)_ = 991, *P* < 0.0001; Fig 5A). Over the 16 unstocked populations sampled in the five different stocked regions, 97.01% ± 0.99% of the fish belonged to local cluster on average while no fish belonged to nonlocal cluster and 2.99% ± 0.99% of the fish belonged to the putatively admixed category (Fig. 5A). In contrast, among the 22 stocked populations, 17.49% ± 6.61% of the fish belonged to the local cluster, 56.48% ± 7.06% of the fish belonged to a nonlocal cluster, and 26.02% ± 3.78% of the fish were putatively admixed (Fig. 5A). Such differences were also observed within each region and were highly significant (Bas-Saint-Laurent: *χ*^2^_(2)_ = 258, *P* < 0.0001; Estrie: *χ*^2^_(2)_ = 163, *P* < 0.0001; Lanaudière: *χ*^2^_(2)_ = 159, *P* < 0.0001; Laurentides: *χ*^2^_(2)_ = 245, *P* < 0.0001 and Outaouais: *χ*^2^_(2)_ = 213, *P* < 0.0001; Fig 5B–F). While we did not observe any significant difference in the proportion of fish assigned to the local cluster, nonlocal cluster or putatively admixed category among the five regions for unstocked populations (*χ*^2^_(8)_ = 8.66, *P* = 0.372), we did observe differences among regions for the stocked populations (*χ*^2^_(8)_ = 311, *P* < 0.0001). In particular, while almost all individuals were assigned to a nonlocal cluster in stocked populations from Estrie, Lanaudière and Bas-Saint-Laurent regions, a noticeable proportion of local fish were detected in stocked populations from Outaouais and Laurentides regions.

### Effects of stocking intensity

Logistic regressions showed that populations' membership to local cluster sharply decreased with the increase of stocking intensity (Fig. 3C,D). With a small stocking intensity of eight fish per hectare (Fig. 3C) or after a single stocking event (Fig. 3D), the logistic regression predicted that 10% of the stocked populations show a 0.00 *local q*-membership value to the local cluster. For a stocking intensity of more than 86 fish per hectare or more than 18 stocking events, the regression predicted that 100% of the stocked populations show a 0.00 *local q*-membership value to the local cluster. For each pair of source and stocked population, a strong positive correlation was observed between the stocking intensity and the proportion of membership of stocked populations corresponding to their source population (*source q*-membership) (Fig. 3E; *ρ* = 0.697; *P* < 0.0001). With <20 fish stocked per hectare, the contributions of source populations were small (*source q*-membership = 0.05 in average). According to the locally weighted scatterplot smoothing (LOESS), the *source q*-membership reached 0.5 with an average of 45 fish per hectare being stocked. However, the contribution of source populations was very variable depending on the pairs of stocked and source populations.

**Figure 6:**
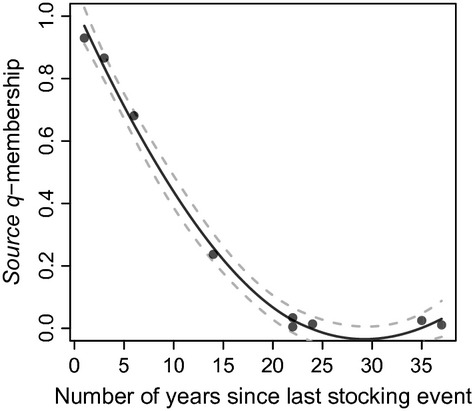
Stocked populations' membership to their source populations cluster (*source q*-membership) as a function of time elapsed (in years) since the last stocking event (N pop. = 9), with a 0.95 confidence envelope.

Finally, there was a negative correlation between time elapsed since last stocking event and stocked populations' *source q*-membership, for each pair of source and stocked population (*ρ* = −0.854; *P* < 0.01; Fig. 6). The LOESS regression predicted that the *source q*-membership decreased from more than 0.95 to 0.20 approximately 15 years following the last stocking event, and that the contribution of the source population was almost null (*source q*-membership < 0.10) 18 years following the last stocking event.

## Discussion

Our main objective was to investigate the impacts of stocking on the distribution of genetic diversity within and among wild lake trout populations over a very large geographic range. Our results demonstrate that stocking resulted in an increase in neutral genetic diversity in stocked populations compared with unstocked ones. Given that stocking practices has traditionally been executed at the administrative regional scale, we observed a decrease in differentiation among populations within regions and an increase in regional genetic differences due to stocking. While unstocked populations were largely composed of local and nonadmixed individuals, stocked populations exhibited variable admixture rates with stocking sources. Furthermore, this study provides a rare clear link between stocking intensity and admixture rates (but see Marie et al. 2010). Finally, our results suggest a decrease over time of admixture in stocked populations after stocking has ceased.

### Effect of stocking on allelic richness in lake trout populations

In line with several previous studies (Ihssen et al. 1988; Halbisen and Wilson 2009; Northrup et al. 2010), genetic diversity (*A*_R_) in unstocked lakes was strongly correlated with lake size (*R*^2^ = 0.41). As lake trout carrying capacity is proportional to the lake area (Shuter et al. 1998), as is the effective population size (*N*_E_; this study and McCracken et al. 2013), variation in population size and *N*_E_ among lakes probably influences the extent of population genetic diversity through variable intensity of genetic drift (Charlesworth 2009; Frankham et al. 2010). Interestingly, we found that the average local genetic diversity (*A*_R_ = 6.16) was more than two times lower than the global diversity (*A*_R_ = 11.75), which is typical of a nonmigratory species where populations are isolated from one another (Waples and Gaggiotti 2006). Overall, genetic drift may have been a determinant process in the erosion of the original genetic diversity, especially given the probable absence or very limited potential for contemporary dispersal among lakes since the end of colonization (6000 years ago; Wilson and Hebert 1996).

The increase of genetic diversity in stocked compared with unstocked lakes as well as the breakdown of the correlation between genetic diversity and lake area for stocked populations reflects the introduction of new alleles from nonlocal populations through stocking. Similar results were reported for brook char (*S. fontinalis*) where stocked populations with a domestic strain also showed a higher genetic diversity than unstocked ones (Marie et al. 2010). In that particular case, stocked populations shared private alleles with nonlocal domestic broodstock used for stocking. Interestingly, it has been shown that when wild local broodstock were used as sources of stocking, no or little difference was observed in the level of genetic diversity following stocking (Eldridge and Killebrew 2008; Small et al. 2009; Gow et al. 2011). In our study, the local increase in genetic diversity was not associated with an increase in the global genetic diversity (*A*_R_ = 11.75 among all unstocked populations, *A*_R_ = 11.05 among the subset of unstocked populations from stocked regions only, *A*_R_ = 11.35 among stocked populations), further indicating a homogenization of the genetic diversity among stocked populations rather than an increase of the global genetic diversity. These results overall confirm that stocking fish from wild but nonlocal origins can significantly modify the extent of genetic diversity of wild stocked populations by increasing local allelic richness through the addition of nonlocal alleles.

### Effect of stocking on genetic structure among populations

Overall, the genetic differentiation among unstocked populations was pronounced (global *F*_ST_ = 0.23) and consistent with other studies on lake trout (Halbisen and Wilson 2009; Northrup et al. 2010; McCracken et al. 2013) as well as with values observed in other nonmigratory salmonids (global *F*_ST_ brook charr 0.23, Marie et al. 2010; bull trout 0.33, Taylor et al. 2001; freshwater Atlantic salmon 0.14, Tessier et al. 1997; European grayling 0.24, Koskinen et al. 2002). Such large genetic differentiation is most probably linked to significant genetic drift and absence or very limited gene flow among populations, as discussed above. In contrast, the twofold decrease in genetic differentiation found among stocked populations (global *F*_ST_ 0.10) compared with unstocked populations indicates a strong genetic homogenization effect induced by stocking at the regional scale. This tendency of genetic homogenization among stocked populations has also been observed in previous studies in several salmonids (e.g., brook charr: Marie et al. 2010; Lamaze et al. 2012; lake trout: Halbisen and Wilson 2009) and is due in all cases to the broad scale practice of using few source populations for broodstock.

Our study also revealed that the genetic differentiation between source and stocked populations decreased with stocking intensity. Reduction of the genetic differentiation between source and stocked population was documented in European grayling *Thymallus thymallus* (Koskinen et al. 2002) and Atlantic salmon *Salmo salar* (Perrier et al. 2011, 2013b) and has been linked to the stocking history and the presence of nonlocal fish and/or putatively admixed individuals. However, these studies did not empirically investigate how stocking intensity may have influenced levels of genetic differentiation between source and stocked populations. Here, we observed that while relatively low-stocking intensities (1–20 fish/ha or 1–4 stocking events) resulted in highly variable levels of genetic differentiation between source and stocked populations, high stocking intensities (more than 40 fish/ha or more than eight stocking events) resulted in a nearly zero genetic differentiation between these groups. To our knowledge, this is the first attempt to empirically determine a threshold value of total homogenization between source and stocked populations. Such a threshold obviously has several implications in terms of management of wild populations as discussed below.

### Artificial hierarchical structuring induced by regional stocking

The weak hierarchical structuring observed among unstocked populations most probably reflects the already evocated influence of genetic drift and the absence of migration among populations leading to a large and not geographically hierarchical differentiation of populations. This observation is concordant with large-scale phylogeography studies of lake trout mtDNA which suggest little contemporary dispersion among populations from distinct mitochondrial lineages since the end of colonization 6000 years ago (Wilson and Hebert 1996, 1998). Besides, Québec's territory was mainly colonized by a single lineage from the Atlantic refuge (Wilson and Hebert 1996) which may have limited potential lineages that could have resulted in hierarchical structuring. Other studies conducted on lake trout also documented little or no evidence of geographical grouping (Halbisen 2008; McCracken et al. 2013). Thus, each unstocked lake trout population could be considered as a unique ‘genetic entity’ without any clear regional grouping.

Considering only stocked populations, a more pronounced genetic differentiation was observed among administrative regions than among lakes within regions, in total opposition with results obtained with unstocked lakes. More precisely, while the genetic variation among stocked populations was four times lower than unstocked, it was two times higher among regions. These results are coherent with the managing strategies used for the last 20 years where stocking has been conducted using one or two broodstock sources to stock lakes from each region. Such a regionally based stocking practice promoted the homogenization of the genetic diversity among stocked population from a same region and consequently caused an artificial increase of a regional based structuring. Breakdown of the genetic structure at various scales as also been observed in other studies where stocking strategies resulted in the homogenization of the genetic diversity among populations (Eldridge and Naish 2007; Perrier et al. 2011).

### Population and individual admixture following stocking

Accordingly with the high genetic differentiation and the low proportion of shared diversity among unstocked lakes, most of the genetic clusters delineated by structure corresponded to a unique lake in the case of unstocked populations. Almost no admixture was found in unstocked lakes in which almost all fish (97.0%) were assigned to the local clusters. These results reinforce the evidence that pronounced genetic drift and the very limited potential for migration among lakes have shaped the distribution of the contemporary genetic diversity in those lake trout populations. These results are concordant with those reported by McCracken et al. (2013) in lake trout as well in other nonmigratory salmonids (Koskinen et al. 2001; Ozerov et al. 2010).

While almost each unstocked lake corresponded to a unique genetic cluster with nearly no admixture, variable admixture rates were observed within stocked lakes as a function of stocking history. Indeed, most of the stocked lakes were highly admixed with their stocking source(s) and most of the individuals were assigned to nonlocal clusters or to the admixed category, while a smaller proportion of trout were assigned to the local cluster. These results demonstrate that stocking with nonlocal fish have caused a genetic displacement of the local gene pool. The occurrence of admixed individuals further indicates that stocked lake trout reproduced with local fish, potentially threatening the local genetic background through introgressive hybridization, as reported in other studies (Eldridge and Naish 2007; Miller et al. 2012). In other salmonids, admixture rates within stocked populations have also been found to be highly variable from almost absence of admixture up to a complete replacement of the native gene pool (Hansen and Mensberg 2009; Perrier et al. 2011). Interestingly, the genetic composition of stocked populations differed among administrative regions and can be related to the stocking modalities of each region. Indeed, in two of five regions, stocking has been conducted less intensively with lower stocking intensity and a mix of local, admixed and nonlocal individuals were identified. In contrast, the three other regions were intensively stocked and stocking was conducted with high-stocking intensity and only nonlocal and admixed individuals were identified in stocked lakes. These results suggest that relatively large and intensive stocking may totally disrupt the original local genetic composition of wild stocked populations.

We acknowledge that using an historical baseline prior to stocking may have allowed a better understanding of the temporal dynamic of admixture (Nielsen et al. 1999; Hansen 2002; Finnegan and Stevens 2008; Metcalf et al. 2012; Perrier et al. 2013a,b2013b), but such samples were not available for stocked lakes. Nevertheless, the genotyping of historical samples from some unstocked populations revealed high temporal stability over 40 years such as was found and discussed for several salmonids (Tessier and Bernatchez 1999; Hansen et al. 2002; Vähä et al. 2008; Ozerov et al. 2010; Gow et al. 2011; Van Doornik et al. 2011) and thus reinforce our interpretation that admixture found in stocked populations most probably resulted from stocking rather than from naturally occurring changes in genetic composition over time. Likewise, the high coherence between the well-documented stocking intensity and the observed admixture of stocked populations reinforces our conclusions.

Stocking intensity largely explained admixture among stocked populations as populations' membership to the local cluster strongly decreased with the increase in stocking intensity. Stocked populations seemed to be highly sensitive to stocking with nonlocal fish as even small stocking intensities (eight fish/ha or a single stocking event) resulted in significant admixture and potential displacement of local gene pool. When stocking intensity increased up to 86 fish per hectare or up to 18 stocking events, all populations were subjected to a total displacement of the local gene pool and therefore to a complete and most probably irreversible loss of the local genetic diversity. These results are in accordance with the few studies which documented stocking intensity to be correlated with the reduction of the genetic integrity in salmonids populations (Eldridge and Naish 2007; Hansen and Mensberg 2009; Marie et al. 2010; Perrier et al. 2013b). However, to our knowledge, this is the first study that clearly links admixture rates to stocking intensity on such a large geographic scale and a wide range of stocking modalities. Nevertheless, many factors may influence degrees of admixture between nonlocal and local fish such as the effective size of the recipient population, the environmental conditions and potential spatial or temporal reproductive isolation (Currat et al. 2008; Hansen and Mensberg 2009; Marie et al. 2012; Perrier et al. 2013b) and require further investigations.

### Admixture decreases following the cessation of stocking activities

Few studies provided clear outcomes about the potential resilience of the original genetic diversity of wild populations after the cessation of stocking (but see Perrier et al. 2013b). Indeed, most studies compared genetic characteristics of the stocked populations between pre- and poststocking samples, which generally highlighted an increase in admixture following stocking (Martinez et al. 2001; Hansen 2002; Susnik et al. 2004; Campos et al. 2007; Eldridge et al. 2009; Karaiskou et al. 2011; Pearse et al. 2011; Glover et al. 2012; Perrier et al. 2013a). Here, while we could not compare samples collected prior or during stocking events, we found that the time elapsed since the last stocking event was a significant factor influencing the current admixture of populations. More precisely, we observed that after cessation of stocking, the genetic membership of stocked populations to their source populations decreased over time to almost reach a null value. Different scenarios could be invoked to explain these results. One scenario could imply genetic drift, where ongoing divergence between source and stocked lakes as the last stocking event would increases with time. Therefore, the longer since the last stocking event, the higher would be the divergence between source and stocked populations. However, the strong genetic temporal stability we found for some unstocked populations is indicative of a low genetic drift in lake trout, which is in accordance with the species long generation time. An alternative scenario would assume that the observed decrease in genetic membership to source population reflects the purge of exogenous genes. This could be linked with a lower fitness of the stocked fish compared to the local ones (Araki et al. 2007a, 2008; Christie et al. 2012; Milot et al. 2013). This could also imply that the impacts of stocking could be reversible if the exogenous genetic components are purged and that local individuals persist, as reported elsewhere (Nielsen et al. 1997; Hansen and Mensberg 2009; Perrier et al. 2013b). In the case of lake trout, such persistence of local individuals may be particularly long since lake trout is a long living species (up to 49 years). Potentially surviving old individuals may thus contribute to the conservation of the formal gene pool even in case of high-stocking intensity. The amount of time required to observe a complete resiliency could of course be variable depending notably on the stocking pressure, the relative amount of local fish persisting over time, potential introgressive hybridization, and fishing pressure (Evans and Willox 1991; Allendorf et al. 2001; Post 2013). However, in the absence of a historical baseline, we cannot confirm either a decrease in genetic membership to source populations, or an increase in local genetic membership, as comparing historical and contemporary samples would have been necessary to test these hypotheses. We also acknowledge that these results could be obtained only for nine pairs of lakes with a low-stocking intensity of 17–22 fish per hectare. Evaluating a wide range of different stocking intensities could have improved our understanding of resiliency processes, which could be different in heavily stocked lakes.

### Perspectives for management

This study demonstrates that stocking profoundly altered the genetic integrity of wild lake trout populations, but that this alteration could be short-lived in some cases with relatively low stocking intensity. Our results on neutral genetic diversity showed genetic introgression to be strongly linked to stocking intensity, however, rates of introgression between different loci under selection could be highly variable as found for other stocked salmonids (Hansen et al. 2010; Meier et al. 2011; Lamaze et al. 2012, 2013). Even though the extent of local adaptations in lake trout remains largely unknown, stocking could alter local adaptation in wild populations, as shown in other fish species (reviewed in Laikre et al. 2010). As preserving local adaptation should be of prime importance in conservation actions, it appears essential to further investigate the extent of local adaptation in lake trout and to quantify potential differences in introgression rates on genes which could confer adaptive advantage or disadvantage. Our results also emphasize the need to manage each lake trout populations independently and to adjust stocking strategies in order to conserve the genetic integrity and putative local adaptations of wild lake trout populations. Our results also provide fisheries' managers with new tools to define conservation targets for unstocked populations as well as for stocked populations. Indeed, a recovery strategy could be worth considering for those stocked populations that did not reach a threshold value of total homogenization with their source, as recovery of the formal gene pool could be possible with time. In the case of the lake trout in the province of Québec, results of this study have been used to propose conservation prioritization based on the genetic status of stocked populations. In addition with other criteria such as the habitat quality, fishing intensity and the population density, genetic ranking of populations has now been integrated in the decision process to optimize stocking strategies in order to reduce the genetic impacts of stocking on wild lake trout populations. Conclusions of this study are clearly applicable for the species in all its distribution range as well as for other freshwater nonmigratory fish that might be highly structured, sensible to overexploitation and subject to stocking.

## Data archiving statement

Data for this study are available from the Dryad Digital Repository: http://dx.doi.org/10.5061/dryad.4cj42.

## Supporting Information

**Table S1.** List, characteristics and GenBank accession number of the markers used.

**Table S2.** Pairwise *F*_ST_ among populations. (spreadsheet).

**Table S3**. Pairwise *F*_ST_ among the subset of lakes for which contemporary and historical samples were available. Number in parentheses is the year samples were collected.

**Table S4.** If stocking occurred, the number of the source lake(s) are indicated in the ‘Source(s) lake(s) used for stocking’ column. A ‘-’ entry, indicates no stocking. (spreadsheet).
