# Supplementary material for: Loss of genetic integrity in wild lake trout populations following stocking: insights from an exhaustive study of 72 lakes from Québec, Canada
Source: Evol Appl. 2014 May 15;7(6):625–44. doi: 10.1111/eva.12160 (PMC4105915; doi:10.1111/eva.12160)
Supplement: Supplementary file 1 — Table S1. List, characteristics and GenBank accession number of the markers used. [file eva0007-0625-SD1.docx]

***Supplementary materials***

**Table S1:** List, characteristics and GenBank accession number of the markers used

| Multi-plex | No | Locus | Reference | Primer sequence (5' to 3') | Dye | Size range (pb) | N | Ta °C | Accession no |
| --- | --- | --- | --- | --- | --- | --- | --- | --- | --- |
| A | L01 | Sfo226*Lav* | Perry *et al.* 2005 | GAGGGCTAGAGACTAGCTTCAG | PET | 364-438 | 35 | 60 | - |
|  |  |  |  | GCAGTGGAACAAATACCCAG |  |  |  |  |  |
|  | L02 | Sfo308*Lav* | Perry *et al.* 2005 | CAGCAATGGGGCTGAAGTAG | HEX | 428-526 | 36 | 52 | - |
|  |  |  |  | GTCACTGTGTGAATCCTCC |  |  |  |  |  |
|  | L03 | SfoD75 | King *et al.* | GTAGTGCCAAAACAGGTAGAGC | HEX | 268-328 | 15 | 52 | AY168197 |
|  |  |  | unpublished | CATCCTTATTCCAACCTCAATC |  |  |  |  |  |
|  | L04 | SnaMSU02 | Rollins *et al.* 2009 | GCCCTTCATTGAGGAACAGA | NED | 184-272 | 24 | 57 | EU331428 |
|  |  |  |  | CTCACACACACGCACAACAA |  |  |  |  |  |
|  | L05 | SnaMSU03 | Rollins *et al*. 2009 | TGGGCAAATTATTGAAGACAAA | FAM | 179-289 | 40 | 57 | EU331429 |
|  |  |  |  | CAGTATACGTCTCTGCCTGTCTG |  |  |  |  |  |
| B | L06 | SnaMSU06 | Rollins *et al.* 2009 | GCTGGTGAGGGAGAGATGAC | NED | 224-352 | 56 | 60 | EU331433 |
|  |  |  |  | CAGCCATGAGAATGGGATTT |  |  |  |  |  |
|  | L07 | Sco202 | Dehaan & | TTGGTTCCTTCCCCTTAGC | FAM | 133-193 | 15 | 60 | AY88871 |
|  |  |  | Ardren 2005 | GCTGAAATAGCCGAATCCA |  |  |  |  |  |
|  | L08 | SnaMSU08 | Rollins *et al.* 2009 | AGAGCAGTCGATTGCAGTAGC | HEX | 118-202 | 21 | 66 | EU331432 |
|  |  |  |  | ACTGCCCTCACTGATGGTG |  |  |  |  |  |
|  | L09 | SnaMSU09 | Rollins *et al*. 2009 | TGATCAGAGATGGCAGTTTCA | FAM | 222-394 | 44 | 66 | EU331439 |
|  |  |  |  | CATTATCCTGGCAACATGGA |  |  |  |  |  |
|  | L10 | SnaMSU10 | Rollins *et al*. 2009 | GCACCTCACCACTCACCTTT | PET | 132-262 | 44 | 63 | EU331434 |
|  |  |  |  | TTATACAGCAGGGCTGAGCA |  |  |  |  |  |
| C | L11 | SnaMSU11 | Rollins *et al*. 2009 | TGATGATGGAAAGGCAGAGG | PET | 213-295 | 43 | 63 | EU331436 |
|  |  |  |  | CCATTTGGGATGCACATACA |  |  |  |  |  |
|  | L12 | SnaMSU12 | Rollins *et al*. 2009 | ATTTTCCACATGCTGCGTCT | FAM | 164-228 | 28 | 63 | EU331437 |
|  |  |  |  | TGAAATAGCTTGGAGCAGTAGC |  |  |  |  |  |
|  | L13 | SnaMSU13 | Rollins *et al.* 2009 | AGTTTCCAAGGCAGCACTGT | NED | 165-327 | 40 | 63 | EU331438 |
|  |  |  |  | TGCTACACAGCAAAATGTGTCA |  |  |  |  |  |
|  | L14 | Sco215 | Dehaan & | GAGAGAGAGAGATGGGTGACA | FAM | 286-314 | 8 | 60 | AY88884 |
|  |  |  | Ardren 2005 | ATCCACAAAACAAGATTGCTA |  |  |  |  |  |
|  | L15 | SnaMSU07 | Rollins *et al.* 2009 | TGCCTATTCCTATTCAATACAGG | HEX | 320-406 | 38 | 60 | EU331435 |
|  |  |  |  | CCCCCACAGTCTGAATCAAT |  |  |  |  |  |
| D | L16 | SnaMSU01 | Rollins *et al*. 2009 | TCACACACCCATTCGTTCAT | HEX | 215-309 | 28 | 57 | EU331427 |
|  |  |  |  | AGCATGGGATAACCACAACC |  |  |  |  |  |
|  | L17 | Sco200 | Dehaan & | GTGCCTTGGTGGAGATTAC | FAM | 178-372 | 44 | 56 | AY88869 |
|  |  |  | Ardren 2005 | CCTTTATGTGTCCCTGTATGA |  |  |  |  |  |
|  | L18 | Smm22 | Crane *et al.* 2004 | CCCAATGCAGATAAGACCTT | NED | 123-221 | 27 | 55 | AY327129 |
|  |  |  |  | TCTATAGGCTTATTTGAATGGAAT | |  |  |  |  |
|  | L19 | SSsp2201 | Paterson *et al.* | TTTAGATGGTGGGATACTGGGAGGC | NED | 274-378 | 27 | 58 | AY081807 |
|  |  |  | 2004 | CGGGAGCCCCATAACCCTACTAATAAC | | |  |  |  |
